# Supplementary material for: Clinical Evaluation of a Line-Probe Assay for Tuberculosis Detection and Drug-Resistance Prediction in Namibia
Source: Microbiol Spectr. 2022 Jun 7;10(3):e00259-22. doi: 10.1128/spectrum.00259-22 (PMC9241941; doi:10.1128/spectrum.00259-22)
Supplement: Supplemental file 1 — Supplemental material. Download spectrum.00259-22-s0001.pdf, PDF file, 0.1 MB [file spectrum.00259-22-s0001.pdf]

Supplement

Table S1: Sensitivity and specificity of GenID® LPA RIF/INH for detection of TB, compared with MGIT culture and/or Xpert® MTB/RIF, for detection of RIF resistance compared to Xpert® MTB/RIF and for INH resistance, compared to culture / INH AST.

	All (n=134)		Cohort 1 (n=79)		Cohort 2 (n= 55)	
GenID® RIF/INH module	sensitivity	specificity	sensitivity	specificity	sensitivity	specificity
Mtb result all patients†	61/82 = 74.4% (63.6-83.4)	51/52 = 98.1% (89.7-100.0)	22/27 = 81.5% (61.9-93.7)	51/52 = 98.1% (89.7-100.0)	39/55 = 70.9% (57.1-82.4)	no observations
Mtb result among smear positive†	56/60 = 93.3% (83.8-98.2)	0/1 = 0.0% (0.0-97.5)	20/22 = 90.9% (70.8.8-98.9)	0/1 = 0.0% (0.0-97.5)	36/38 = 94.7% (82.3-99.4)	no observations
Mtb result among smear negative†	5/22 = 22.7% (7.8-45.4)	51/51 = 100% (93.0-100)	2/5 = 40.0% (5.3-85.3)	51/51 = 100% (93.0-100)	3/17 = 17.6% (3.8-43.4)	no observations
RIF resistance among Mtb +‡§	32/38 = 84.2% (68.7-94.0)	19/19 = 100% (82.4-100)	0/1 = 0.0% (0.0-97.5)	18/18 = 100% (81.5-100.0)	32/37 = 86.5% (71.2-95.5)	1/1 = 100% (2.5-100)
INH resistance among Mtb +‡	26/29 = 89.7% (72.6-97.8)	22/24 = 91.7% (73.0-99.0)	2/3 = 66.7% (9.4-99.2)	17/18 = 91.4% (72.7-99.9)	24/26 = 92.3% (74.9-99.1)	5/6 = 83.3% (35.9-99.6)

† Mtb detection is based on Xpert® MTB/RIF or culture (BACTEC® MGIT 960) as reference standard, Mtb detection of LPA was assessed in the GenID® RIF/INH module

‡ RIF resistance declared, when rpoB mutation present or rpoB wildtype missing, INH resistance declared, when inhA and/or katG mutation present or inhA and/or katG wildtype missing

§ RIF resistance is based on Xpert® MTB/RIF as reference standard, INH resistance is based on culture/ AST as reference standard

AST – antimicrobial susceptibilty testing by culture; Mtb - Mycobacterium tuberculosis; INH - Isoniazid; RIF - rifampin

Table S2: Frequency of LPA resistance results by loci on GenID®LPA RIF/INH, FQ and SLI strip in patients with overall interpretation “resistance detected” in DST and LPA.

		Number of LPA results for locus			% LPA results for locus	
**Drug**	**locus**	**tested**	**not resistant**	**resistant**	**% not resistant**	**% resistant**
**INH**	inhA wt missing	26	12	14	46.2	53.8
	inhA mut	26	16	10	61.5	38.5
	katG wt missing	26	11	15	42.3	57.7
	katG mut	26	11	15	42.3	57.7
**RIF**	RpoB wt 513-516 missing	19	16	3	84.2	15.8
	RpoB mut 516	19	19	0	100.0	0.0
	RpoB wt 522-526 missing	19	15	4	78.9	21.1
	RpoB mut 526	19	16	3	84.2	15.8
	RpoB wt 529-533 missing	19	7	12	36.8	63.2
	RpoB mut 531	19	7	12	36.8	63.2
**STR**	rpsL 43 wt missing	10	1	9	10.0	90.0
	rpsL mut K43R	10	2	8	20.0	80.0
	rpsL 88 wt missing	10	8	2	80.0	20.0
	rpsL mut K88R, K88Q	10	9	1	90.0	10.0
	rrs 513-517 wt missing	10	10	0	100.0	0.0
	rrs mut 513-517	10	10	0	100.0	0.0
**SLI**	rrs 1401,1402 wt missing	3	0	3	0.0	100.0
	rrs mut A1401G	3	0	3	0.0	100.0
	rrs mut C1402T	3	3	0	100.0	0.0
	rrs mut C1484C/T wt missing	3	3	0	100.0	0.0
	mut rrs C1484C/T	3	3	0	100.0	0.0
**FQ**	gyrA 90,91,94 wt missing	6	0	6	0.0	100.0
	gyrA mut A90V	6	6	0	100.0	0.0
	gyrA mut S91P	6	6	0	100.0	0.0
	gyrA mut D94A	6	5	1	83.3	16.7
	gyrA mut D94N	6	5	1	83.3	16.7
	gyrA mut D94Y	6	6	0	100.0	0.0
	gyrA mut D94G	6	1	5	16.7	83.3
**EMB**	embB 306 wt missing	11	0	11	0.0	100.0
	embB mut M306V	11	8	3	72.7	27.3
	embB mut M306I; G918A	11	5	6	45.5	54.5
	embB mut M306I; G918C	11	8	3	72.7	27.3
	embB mut M306I; G918T	11	11	0	100.0	0.0
